# Snakebite Clinics and Pathogenesis: From Preclinical to Resource Mapping Studies

**DOI:** 10.3390/toxins15110626

**Published:** 2023-10-25

**Authors:** Manuela B. Pucca, Hui F. Wen, Ana M. Moura-da-Silva, Wuelton M. Monteiro

**Affiliations:** 1 Department of Clinical Analysis, School of Pharmaceutical Sciences, São Paulo State University, Araraquara 14800-903, Brazil; 2 Butantan Institute, São Paulo 05503-900, Brazil; fan.hui@butantan.gov.br (H.F.W.); ana.moura@butantan.gov.br (A.M.M.-d.-S.); 3 Department of Medicine and Nursing, School of Health Sciences, Dr. Heitor Vieira Dourado Tropical Medicine Foundation, Manaus 69040-000, Brazil; 4 Department of Teaching and Research, Dr. Heitor Vieira Dourado Tropical Medicine Foundation, Manaus 69040-000, Brazil

Amidst the global healthcare landscape, the menace of snakebite envenoming (SBE) has persisted, silently afflicting millions and annually claiming tens of thousands of lives. Indeed, in 2017, the World Health Organization (WHO) reclassified snakebite envenoming as a Category A Neglected Tropical Disease (NTD), finally prompting worldwide recognition of the profound health and economic devastation caused by these venomous encounters. Then, in 2019, WHO unveiled an ambitious strategy: to slash snakebite envenoming-related mortality and disability by 50% before 2030 [1,2]. This editorial marks the inception of our Special Issue, “Snakebite Clinics and Pathogenesis: From Preclinical to Resource Mapping Studies”, which stands as a guiding light in our collective effort to confront SBEs. Gathering insights from research on snakebite envenoming outcomes, diagnostic advancements, uncommon case reports, therapeutic strategies, and healthcare professional training, this Special Issue is dedicated to disseminating knowledge and charting a course towards a future where snakebite envenomings cease to be a neglected tragedy and evolve into a preventable and manageable challenge.

In their pioneering study, Murta et al. [3] explored the experiences of healthcare professionals (HCPs) providing medical care to indigenous people with SBEs in the Brazilian Amazon. They conducted group discussions during a three-day training session for HCPs from the Indigenous Health Care Subsystem, involving 56 participants split between Boa Vista (Roraima) and Manaus (Amazonas), which are state capitals located in the Brazilian Amazon Forest. The study revealed three key findings: indigenous people are open to receiving antivenom but prefer not to leave their villages for hospitals; HCPs require antivenom and additional resources to improve patient care; and HCPs advocate for a collaborative, culturally sensitive approach to SBE treatment. To address these challenges, the study suggests decentralizing antivenom distribution to local health units. However, the diverse ethnicities in the Brazilian Amazon pose a challenge, necessitating further research on preparing HCPs for intercultural contexts.

Even when antivenom is available in low-resource areas, health workers do not receive adequate training to manage SBEs. The study of Rocha et al. (2022) [4] aimed to develop and validate a clinical practice guideline (CPG) for SBE management across Brazil. Content validation was performed by a panel of expert judges with academic and/or technical expertise in SBE management, and semantic validation was performed by analyzing focus group discussions with doctors and nurses from three municipalities of the Brazilian Amazon. This study presents the successful development and validation process of a CPG for SBE management, which is targeted to a specific low-resource, high-burden setting. This development and validation process can be adapted to other settings and/or other neglected tropical diseases. In the health system domain, this strategy involves ensuring the production and distribution of safe and effective antivenom treatment and strengthening local health systems.

Bhatia et al. (2022) [5] highlight that there is an urgent need to replace the excessive use of animals in snake antivenom production. We tested the efficacy of a single batch of polyvalent antivenom from bioproducts limited to *Echis carinatus* venom collected from Tamil Nadu, Goa, and Rajasthan, using different in vitro assays. The use of both binding and functional assays allowed us to measure the efficacy of the antivenom. By normalizing the scale of measurements of the neutralization capacity of the Indian polyvalent antivenom using different in vitro assays, we were able to arrive at an efficacy score for *Echis carinatus* venoms that could be used to predict the ED50. This approach captures the variation in venom toxins shown by snake species and paves the way to replace the use of mice for evaluating antivenom potency.

*Protobothrops mucrosquamatus* snakebites are frequent in Taiwan, and the species’ widespread distribution and diverse habitats drove Chiang et al. (2022) [6] to investigate envenoming effects and relevant venom variations. The results showed minor differences in the protein family, with variations in acidic phospholipases A_2_s, serine proteinases, metalloproteinases, C-type lectin-like proteins, and other less abundant components. Moreover, clinical manifestations of envenomed patients hospitalized in northern Taiwan revealed differences in local symptoms, such as ecchymosis and blistering. The mechanism of these local effects is probably related to the venom components’ geographical variability. These findings will help to improve the management of *P. mucrosquamatus* bites in Taiwan.

Vera-Palacios et al. (2022) [7] investigated in vivo the ability of *Urospatha sagittifolia* (Araceae) to modulate the catalytic activity of *Bothrops atrox* venom, and their toxic consequences, such as edema, skin hemorrhage, and lethality. Ethanolic extract, which is rich in phenolics, alkaloids, coumarins, and flavonoids, reduced these three parameters. The authors concluded that these findings will support future studies with purified metabolites as new agents for the treatment of *B. atrox* snakebites, an important public health problem in the Amazon region.

The study by Manson et al. [8] marks a groundbreaking leap forward in the realm of SBE treatment, with a particular focus on combating the toxicity of Three-Finger Toxins (3FTxs) of *Naja ashei* snake venom. These potent venom-derived toxins are prevalent in *N. ashei* venom and have posed a formidable challenge to effective antivenom therapy. What sets this research apart is the development of monoclonal antibodies (i.e., P4G6a, P6D9a, and P6D9b) meticulously designed to target these troublesome 3FTxs. Remarkably, the monoclonal antibodies demonstrated exceptional binding capabilities to the target 3FTxs, outperforming even the leading commercial antivenoms available in the Kenyan market. The true breakthrough lies in the combined use of these monoclonal antibodies, where their cocktail exhibited superior toxin inhibition compared to traditional antivenoms.

Alsolaiss et al. [9] sheds essential light on the complex and diverse acute responses triggered by African snake venoms, a critical aspect of understanding the pathophysiology of SBEs. Using a well-designed murine model, the research systematically evaluated the acute-phase and inflammatory reactions induced by ten different African snake venoms, with a particular focus on sub-Saharan African species, including the spitting cobra (*Naja nigricollis*) and forest cobra (*N. melanoleuca*), as potent inducers of acute-phase and inflammatory responses, with *N. nigricollis* venom stimulating a remarkable 100-fold increase in systemic interleukin 6 (IL-6). Moreover, the study revealed species-specific changes in red blood cell morphology, lymphopenia, neutrophil leukocytosis, and marked hemolysis and platelet aggregation levels in response to these venoms. These findings underscore the intricate and diverse nature of acute responses to envenoming, paving the way for potential diagnostic and therapeutic advancements that could greatly benefit snakebite victims.

A very interesting review was also presented in the Special Issue. Huang et al. [10] analyzes 35 cases of snakebites, primarily from front-fanged snakes, like vipers and cobras, as well as a few rare instances from rear-fanged snakes. Viper bites often result in severe complications, such as ischemic strokes and intracranial hemorrhages, leading to fatalities in some cases. In contrast, elapid bites are primarily manifested as neural, cardiac, and ophthalmic disorders. Remarkably, rear-fanged snakebites, characterized by shallow bites and minimal venom injection, rarely cause severe complications. An essential takeaway from the review is the pivotal role of antivenom (AV) treatment, although it also discusses various therapeutic agents that could potentially complement AV treatment for snakebite-induced complications. 

Furthermore, the Special Issue delved into two unconventional snakebite case reports, one conducted in Romania and the other in Brazil, subjecting them to comprehensive examination and discussion. Nitescu et al. [11] offers a unique perspective on snake envenomation, focusing on a specific exception within the European viper (*Vipera berus*) species. While most *V. berus* bites typically lack neurotoxic effects, their study highlights rare cases involving subspecies found in the Carpathian Basin of southeastern Europe that do induce such symptoms. The study presents a compelling case of a 5-year-old girl from southern Romania who experienced neurotoxicity, alongside systemic and local symptoms, following a bite from one of these Carpathian Basin *V. berus* subspecies. This case provides pivotal insights, affirming that venom from *V. berus* subspecies in the Carpathian Basin region can indeed induce neurotoxic effects. Additionally, it underscores the effectiveness of monospecific antivenom treatment in rapidly and completely mitigating the envenomation’s effects, offering valuable clinical guidance for the management of such rare cases. In contrast, Oliveira et al.’s [12] case report delves into the often-overlooked long-term musculoskeletal disabilities resulting from snakebites in indigenous communities in Brazil. The report focuses on a 32-year-old male indigenous patient envenomed by a *Bothrops* species (lancehead snake), highlighting the significant and enduring health challenges posed by snakebites. Over approximately 2 years and 6 months, the patient underwent various medical interventions, including debridement, tissue reconstruction, and physical therapy, resulting in improved mobility but a lasting impact on his gait. This case report emphasizes the need for a comprehensive healthcare approach, including physiotherapy, plastic surgery, orthopedics, and social support, to aid in the reintegration of snakebite survivors into their communities.

Antivenom treatments for SBE patients have existed for more than 130 years, remaining the only therapeutics available for this neglected problem. Remarkably, despite advances in the health system, access to antivenom treatment is poor in most areas of low-income countries. Better logistics for the transportation of antivenoms and other commodities is an issue to be addressed, as well as realistic and comprehensive health programs. In parallel, many investments are still needed for the research and development of more effective antivenoms for some species of snakes, as well as for the advance of small-molecule inhibitor-based drug therapies.
